# A proposed framework for practical multimodal management of osteoarthritis in growing dogs

**DOI:** 10.3389/fvets.2025.1565922

**Published:** 2025-04-28

**Authors:** Denis J. Marcellin-Little, Donald A. Hulse, Janice L. Huntingford, Tamara Grubb, Matthew W. Brunke, Arielle Pechette Markley, Bethany Frank

**Affiliations:** 1 Department of Surgical and Radiological Sciences, School of Veterinary Medicine, University of California, Davis, Davis, CA, United States; 2 College of Veterinary Medicine, Texas A&M University, College Station, TX, United States; 3 Essex Animal Hospital, Essex, ON, Canada; 4 Department of Veterinary Clinical Sciences, Washington State University, Pullman, WA, United States; 5 Veterinary Referral Associates, Gaithersburg, MD, United States; 6 Red Sage Integrative Veterinary Partners, Fort Collins, CO, United States; 7 Vetoquinol United States, Fort Worth, TX, United States

**Keywords:** osteoarthritis, dog, physical rehabilitation, weight management, non-steroidal anti-inflammatory drugs, nutraceuticals, nutritional supplements, medical management guidelines

## Abstract

Osteoarthritis (OA) is a ubiquitous problem affecting dog joints, particularly the hip, elbow, stifle, and spine. OA most often results from developmental orthopedic problems such as hip dysplasia, elbow dysplasia, and patellar luxation and from injuries to the cranial cruciate ligament. Several management approaches have been proposed to manage OA, including steps to modulate growth, physical activity, and exercise, nutrition and nutritional supplementation, medications, physical rehabilitation, and surgical procedures. This article is the first in a series of articles that propose steps for practical OA management in dogs at various life stages. The review presented here focuses on growing dogs. The text describes the early pathophysiology and diagnosis of OA. The physical, nutritional, analgesic, and surgical management options of OA in growing dogs are presented. The application of these management options is described for three dogs. The overall approach to the management of OA in growing dogs is discussed.

## Introduction

Clients wish for a full and unrestricted life for their dogs. Osteoarthritis (OA) threatens their quality of life because it is the most common orthopedic condition observed in dogs and it is often associated with severe chronic joint pain (1). In a large study of more than 450,000 dogs, the annual period prevalence of OA was 2.5% (2). In that study, risk was increased when dogs were neutered, heavier, and older than 8 years.

The impact of OA in dogs can be physical, behavioral, and social (3). Physically, limb use, mobility, and function during daily activities can be compromised (4). Behaviorally, dogs with chronic pain often express problem behavior (5). Socially, OA can create major life challenges for the owner-animal bond because it negatively impacts the owners’ lives, including creating physical and mental hardships and financial challenges (6). The career longevity of working and sporting dogs with OA is also negatively impacted (7–10). The lifespan of dogs with OA is also shortened by approximately 2 years (11).

No single solution can be used to manage or cure OA and its impact. However, practical management steps and whole-body wellness can positively impact the life of dogs with OA. Whole-body wellness for dogs can be defined as increased owner mindfulness about general aspects of their dog’s health, including the dog’s lifestyle, nutrition, and exercise (4, 12). Whole-body wellness is relevant at all life stages: puppyhood, adulthood, and life as senior dogs. Beyond its positive impact on the life of all dogs and their owners, whole-body wellness enhances the management of OA. This is because lifestyle, nutrition, and exercise lead to increased owner awareness of their dog and increased dog fitness and strength. For dogs with OA, a multimodal management plan is more effective than any single management step (13). Multimodal therapy can include whole-body wellness, physical rehabilitation, nutraceutical and supplements, and pharmaceuticals, as well as surgical intervention, when appropriate (14). Pharmaceuticals may be administered orally, systemically or intraarticularly depending on the stage of OA and the specific needs of the patient.

This review presents practical guidance for preventing and managing OA in dogs during the first life stage. The text describes and discusses the use of all OA management options, including physical, nutritional, and analgesic management of OA in growing dogs. The application of these management options in 3 classic cases is also included.

## OA pathophysiology

Canine osteoarthritis (OA) is a common problem in dogs characterized by irreversible and progressive degenerative changes to joints leading to pain and disability (15). The prevalence of OA in dogs is not known. An estimated OA prevalence of 20% is reported in the literature, based on the results of a survey of 200 veterinarians conducted in 1996 (15). OA is a complex syndrome that can result from mechanical and biological factors. It has been described as the consequence of abnormal forces applied to joints with normal physiology or from normal forces applied to joints with abnormal physiology (16). OA includes inflammation (17), the degeneration of articular surfaces, changes in the synovial membrane (18), changes in subchondral bone (19), and bone production at the edges of the articular surface and at the origin of intraarticular tendons (20). The combination of these changes leads to loss of joint function and disability. Loss of joint function may include pain, changes in joint alignment, and changes in joint motion. Disability may result from chronic pain (21), loss of strength (22), loss of fitness, and loss of mobility resulting from the loss of joint function (23). The progression rate and clinical signs of OA in dogs vary widely. Clinical signs can be severe at a young age in some dogs.

Overall, the discovery of OA in growing dogs is relatively uncommon because OA is seldomly detected based on its common triggers (joint subluxation or joint instability). Instead, OA appears to be discovered because of the synovial or subchondral bone inflammation that results from joint subluxation and instability. Inflammation leads to pain. Acute pain leads to limb disuse and chronic pain (16). Chronic pain leads to a long-term physical and functional signs, including a change in demeanor (loss of playfulness, increased introversion, loss of willingness to exercise), a loss of limb function, and in severe cases, a loss of mobility (24). Subjectively in growing dogs, the signs of OA are most often linked to inflammation, acute pain, and limb disuse.

## Diagnosis of OA

The early diagnosis of OA in dogs is challenging but important. The early diagnosis of OA in dogs presents key advantages over a late diagnosis. From a pathophysiologic viewpoint, the inflammatory component of OA may be severe in the early stage of the problem. An early diagnosis of OA brings the opportunity to alleviate joint inflammation. This may limit the progression of the problem. The diagnosis of OA most often relies on the presence of osteophytes and enthesophytes (new bone formation at the insertion site of musculoskeletal soft tissues). However, in puppies with joint disease, the main abnormal finding in the joint is often joint subluxation. Subluxation often leads to the development of OA within weeks to months (25, 26). Therefore, it is logical to diagnose clinical OA as soon as the presence of subluxation is observed.

Some forms of OA appear to be more readily diagnosed than others. For example, OA from an osteochondral fragment in the stifle or tibiotarsal joint is likely to be diagnosed promptly because of severe lameness and joint effusion. Other forms of OA are less likely to be diagnosed promptly. Hip dysplasia in dogs with transient hip subluxation and bilateral elbow dysplasia may not have a severe impact on gait in the short term and may not be readily confirmed on radiographs because hip subluxation is intermittent and because elbow subluxation may be impossible to document on radiographs before the development of osteophytes. Delays in the identification of OA may reflect a certain lack of attention to the dog’s gait and joint comfort. The use of the Ortolani sign during hip joint palpation may be required to diagnose hip subluxation, before the onset of hip OA. For dogs with patellar luxation, the OA diagnosis may be delayed when the luxation is more intermittent. Dogs with severe luxation are readily identified because they may be non-weight bearing (unilateral luxation) or may have a crouched posture and limited use of their pelvic limbs (bilateral luxation). Conversely, dogs with milder forms of patellar luxation are diagnosed later in life and have more severe OA than younger dogs with patellar luxation (27).

From a therapeutic viewpoint, clinical signs are much more limited in early OA compared to chronic OA and are, therefore, easier to control. Pain in early OA is most often acute rather than chronic. The impact of pain, referred to in pain physiology as the *neuropathobiological signature of pain*, is simpler and more limited in acute pain than chronic pain. Acute pain is focal. Chronic pain is focally more severe, due to local sensitization and allodynia. Central pain is present due to *central sensitization* (28). Practically, the simpler neuropathobiological pain signature means that fewer drugs and fewer non-drug-based pain-relieving steps (such as manual therapy and electrophysical modalities) will likely be required to control pain and medical therapy will be required for a shorter period. Pain may reemerge after the end of medical therapy and pulsed medical therapy may be required. Also, mobility and function are rarely compromised in puppies with OA. Therefore, ambulation assistance and advanced therapeutic exercises such as underwater treadmill therapy are unlikely to be needed when OA is diagnosed early and managed promptly. The opportunity to modulate growth by adjusting food intake, to control the timing of neutering, and to prevent excessive weight gains are other key advantages of the early diagnosis of OA in dogs. Also, and subjectively, owner education regarding OA pathophysiology and management can be more progressive when the disease is diagnosed early. Early OA and its management are simpler than late-stage OA for owners to understand and manage. It may also be easier to adopt a healthy long-term exercise strategy when OA is diagnosed in a puppy because bad habits may be harder to break, and good habits may be harder to learn when OA is diagnosed or managed later in life.

## Whole body wellness

Whole-body wellness is important in puppies, it requires regular physical examinations, deworming, protection against ectoparasites and close monitoring of growth and body weight (29). It is critically important not to overfeed puppies because overfeeding (i.e., excess energy intake) accelerates growth and promotes excess weight, leading to an increase in the severity and impact of OA in dogs (30). Increased body weight could promote the progression of OA through multiple mechanisms, it increases the forces resisted by joints, it alters joint motion, and it leads to a systemic inflammatory response (31, 32).

Studies have demonstrated that rapid weight gain during the first year of life is detrimental to skeletal development (33–35). Neutering (ovariohysterectomy or castration) should be discussed, particularly when a puppy has OA because of a genetic disease, like hip or elbow dysplasia, and should therefore not be bred. Neutering offers clear health benefits to dogs, including a lower risk of developing several tumor types (36). However, neutering is also associated with an increased risk of weight gain after surgery (29) and with an increased risk of being diagnosed with orthopedic problems such as a cruciate ligament injury (37) or OA (38). The mechanisms linking neutering and the risk of OA are not clear. They may be mediated by excess weight, hormonal changes, or other mechanisms. Consequently, there is no optimal age to neuter dogs and cats. The decision is based upon breed, body size, predisposition to specific diseases, and other factors (39).

Because OA progresses more rapidly in overweight dogs, the prevention of excess weight is key to lowering the likelihood of rapid OA progression (24). Food plays a complex role in the relationship between owners and their pets (40, 41), it is therefore important to set up a healthy nutrition pattern in puppies (42). Also, because weight loss programs do not always succeed, an emphasis must be placed on avoiding the initial gain of excess weight. A large-scale study of client owned puppies showed that puppies younger than 6 months had energy intake requirements that were approximately 80% of the 2006 National Research Council (NRC) recommendations and approximately 88% of the NRC recommendations in older puppies, suggesting that the NRC guidelines may overestimate nutritional needs (30).

Multiple complete and balanced diets exist for the feeding of puppies. Despite this, some owners elect to make their pet’s food at home often without the consultation of a veterinarian, or veterinary nutritionist. Home prepared diets carry the risk of nutrient insufficiencies, excesses, and imbalances (43). This is particularly concerning for large breed puppies who are at risk of developmental orthopedic disease (DOD). Hip dysplasia and osteochondrosis make up many musculoskeletal diseases, with a possible nutritional etiology reported in the literature (43, 44). The most critical period for the development of DOD occurs before epiphyseal closure during the growth phase. Risk factors that increase the risk of DOD in young dogs include unrestricted feeding, overfeeding high energy foods, feeding foods, treats, or supplements with excess calcium, and being a large or giant breed (43).

Puppies have higher protein, essential fatty acid (EFA) and mineral requirements (44). High protein diets that have been balanced for large breed puppies do not negatively affect skeletal development but will mildly affect growth rate (44). Increased levels of docosahexaenoic acid (DHA) can improve retinal development, learning, and memory in puppies. Particularly for large breed puppies, it is recommended to feed a diet that controls calcium and calorie intake and maintains a 2:1 to 1:1 calcium to phosphorus ratio. Unbalanced diets, diets with too much or too little calcium, or all meat diets without bone elements predispose puppies to DOD, osteopenia, and pathological fractures. In general, large-breed puppy food should contain a range of 2.0 to 4.5 g calcium/1000 kcal (45). Virtually all commercial diets will contain this minimum amount of calcium; however, some all-life stage diets contain significantly more than the maximum. Although when reading a bag or can of dog food, adult foods look similar to many large breed puppy formulations, however, feeding adult maintenance diets to puppies is not recommended as these foods will be a deficient in trace minerals or vitamins or have excessive calcium that may affect growth (44).

When feeding large or giant breed puppies, the goal should be moderate energy restriction. From weaning to 4 months, puppies should be fed three times their resting energy requirement (RER), then two to 2.5 times the RER until 6 to 12 months of age, depending on the breed (45). In general, most puppies kept in good body condition will escalate calorie consumption until 4 to 6 months of age at which time they reach peak caloric consumption and rarely need more calories to complete proper growth to adulthood (45, 46). This escalating caloric consumption likely extends to 8 to 10 months in giant breed dogs. All breeds can be switched to adult maintenance formulas at 1 year of age. *Ad libitum* feeding and overnutrition contribute to the development of OA, as this places excess stress on potentially genetically abnormal joints (43, 44). Controlled caloric intake should continue for life of the dog to avoid abnormal stresses on joints.

Maintenance of lean body weight throughout the pet’s life, starting with the growth phase, is critical for longevity and delaying the development of OA. A 14-year lifespan study in Labrador Retrievers showed that, when fed to maintain a lean body condition from puppyhood, and throughout life, dogs lived on average 1.8 years (15%) longer, than their free fed littermates (4, 11). Maintaining optimal body condition throughout life can delay the onset and reduce the severity of OA in dogs. For example, in the lifelong study of 7 litters of Labrador retrievers, when dogs were evaluated for the presence of OA in the shoulder and hip joints, 68% of the heavier dogs had hip or shoulder OA compared to 10% of slender dogs. Also, 77% of the heavier dogs had OA in 2 or 3 joints (among their shoulders and hips) compared to 10% of the slender dogs (24). Lean dogs also showed delayed onset of other chronic and age-related diseases (24). Scoring body condition using a 9-point or 5-point scale is done routinely in practice (47, 48). However, veterinarians (and owners) may underestimate body condition (49, 50) or may decide to overlook excess weight to avoid a challenging conversation with owners. Societal norms and the standards for certain dog breeds make it more challenging to keep dogs slender, complicating the issue for dogs with OA.

In early OA, clinical signs are relatively discreet and mostly result from acute joint inflammation and pain. Clinically and subjectively, dogs with early OA usually do not have a loss of muscle mass or loss of joint motion, unlike dogs with chronic OA (51). The signs of early OA tend to be more transient and easier to control than the signs of chronic OA. Osteoarthritis in growing dogs often results from hip or elbow dysplasia. In the hip, early OA most often results from hip laxity, subluxation, and secondary joint inflammation, followed by focal cartilage damage to the dorsal acetabular rim and femoral head. In the elbow, the trigger of early OA is less clear. Some attribute it to abnormal geometry of the ulnar notch and others to radioulnar subluxation. Both abnormalities lead to damage to the articular surface of the ulna and, to a lesser extent, to the medial aspect of the humeral condyle. Elbow subluxation and secondary elbow OA are also seen in chondrodystrophic dogs that have impaired growth of their long bones, particularly in the antebrachium (52). Less often, OA may result from the presence of an osteochondral fragment in a joint, such as, with osteochondritis dissecans or because of an intraarticular fracture. Clinically, it appears that OA is more likely to be detected in puppies when dogs are larger, are affected in multiple limbs, or are severely affected because the negative impact of OA is more immediate and severe in these situations. The detection and management of OA in puppies, however challenging, is particularly important because it is more effective to preserve joint health than to attempt to recover it once it is lost. Also, chronic joint inflammation and chronic joint pain have complex physiologic and physical consequences that may only be partially reversible.

In conclusion, OA in the growing dog is most often caused by hip or elbow dysplasia. Signs of acute OA are more transient and more responsive to conservative multimodal therapy than signs of chronic OA. When managing OA, multimodal management is more effective than any single management strategy. Nutritional errors and imbalances promote the development of OA in dogs. Maintaining lean body condition delays the onset and slows the progression of OA. It also increases longevity and is essential for overall quality of life.

## Physical wellness

A healthy daily routine should be established for each puppy based on their general demeanor and their future purpose as a pet, working dog, or sporting dog. A consistent daily routine will maximize training and compliance and will minimize fear, frustration, and anxiety. The implementation of a healthy daily routine takes time, effort, and consistency. Success is more likely when owners develop a long-term relationship with a trusted caregiver and advisor (53).

For growing dogs with OA, the initial clinic visit is the foundation of a long-term relationship with the owners and the beginning of long-term OA management. In dogs at risk of OA, it is wise to set aside time to gather information from owners and to discuss OA management with owners. Information about the dog’s functional ability is collected using one or more validated client questionnaires such as the Canine Brief Pain Inventory (CBPI), the Liverpool OsteoArthritis in Dogs (LOAD), the Helsinki Chronic Pain Index, and the Canine Orthopedic Index (COI) (54–57). Information about function particularly as it relates to daily activities (for example, the ability to climb a set of stairs or perform a small jump) is also collected during the period of observation preceding the physical examination. Functional information is critically important because it serves as a baseline for future evaluations of disease progression and response to therapy (13). A set of time- and place-specific daily activities can be used to develop a set of client-specific outcome measures (CSOM). Just like standard validated questionnaires, this CSOM set will be used to monitor response to therapy and disease progression. A change in any of the scores is suggestive of a change in limb function or pain, with a proportionality between the amplitude of the score change and the amplitude of the change in pain perceived and function. The minimal clinically important difference (MCID) of a questionnaire score can be determined. Rather than being a numerical change with unclear clinical impact, the MCID is the score difference that represents a clinically impactful change: a clinically significant positive response to therapy or a clinically significant increase in disease severity. In one study, the MCID for the LOAD and the COI, were 4 and 14, respectively (58). Notably, none of the client questionnaires were designed to evaluate function and disability in puppies and questionnaires are not currently validated when used to evaluate puppies (59, 60).

Dog owners should understand what joints and limbs are affected by OA, what physiologic, physical, and functional responses are observed at the time of evaluation and anticipated in the future. Changes in demeanor (e.g., a loss of playfulness) and mobility (e.g., a loss of ability to jump into a motor vehicle or a loss of willingness to go on a walk) are particularly important, as they are likely surrogate markers of the presence of chronic pain (61, 62). Observing for changes in demeanor (playfulness) is particularly relevant in puppies with OA because the problem is acute and recent. However, loss of function (mobility) is less likely because OA rarely alters strength or joint motion in the near term.

A better client understanding of the impact of OA on dogs will help clients notice changes more promptly when signs recur after therapy or when new signs emerge. Clients can then report these changes to the clinical team immediately. In turn, the clinical team will be able to provide advice or therapy more rapidly. The owner and caregiver should be aligned on the owner’s preferred approach to manage OA. This includes the owners’ level of involvement, the willingness to try therapeutic options supported by varying levels of scientific evidence, the cost of care, whether care is delivered by owners or medical professionals, and what to do when facing a symptom flare. In other words, care should be patient centered rather than clinician centered (53). To facilitate contact with owners, communications should consistently take place with the same clinical team who know the dog and the owner. Veterinary technicians make great case coordinators.

The foundation of OA management includes appropriate nutrition, regular leash walks and play, and a safe and effective pain-relieving strategy when joint pain is observed. Intensity, duration, and frequency should match patients’ needs for all exercises. Exercise programs often start with slow walks lasting 5 to 10 min and happening 3 to 5 times a week. If dogs do well, the duration, the intensity, and the frequency increase progressively and additional exercises are introduced (63). When dogs experience a symptom flare, exercise is curtailed while pain is managed.

In conclusion, establishing a relationship and good rapport between the owner and the clinical team is critical to the long-term successful management of OA. Client questionnaires and pain scales can be used to determine the patient baseline and track treatment response and disease progression. A consistent daily routine will maximize training and compliance and will minimize fear, frustration, and anxiety.

## Nutritional supplements

Several nutritional supplements are commonly used by dog owners for the prevention or treatment of OA. Nutritional supplementation is one of the areas of pet care growing most rapidly. Supplements may provide nutritional or therapeutic benefits. They include vitamins, minerals, amino acids, enzymes, herbals, or botanicals. Dietary supplements may be added to food to make it nutritionally complete or to balance the diet. A nutraceutical is a therapeutic supplement that provides potential medical or health benefits.

In general, puppies eating a reputable, complete, and balanced diet need few supplements. B-vitamins, digestive enzymes, probiotics, or antioxidants may be needed during times of stress. For puppies with OA, proactive management using nutrition or supplements starting in puppyhood is often recommended. Many clients are interested in preventing the progression of OA in their puppies, particularly when their breed or conformation suggests an increased risk of OA, when they are intended to become a sporting dog, or when they already show signs of OA. Puppies can be divided into three groups: healthy with low OA risk, healthy with high OA risk, and early OA onset.

Healthy puppies with conformational abnormalities, puppies who will engage in high-intensity athletic activity, and puppies from breeds known to get early onset OA are considered high risk for developing OA. In puppies with OA, joint supplementation aims to lower inflammation and mitigate the development of OA, working synergistically with pain medications. When managing OA, nutraceuticals have multiple physiologic benefits, such as decreasing inflammatory prostaglandins (PGE_2_), decreasing the production of matrix metalloproteinases (MMP-2, MMP-9) responsible for cartilage damage, and increasing the concentration of tissue inhibitor of MMP (TIMP2) (64). Nutritional supplements should be made of high-quality ingredients. Quality can be ensured through certification, for example through certification by the National Animal Supplement Council (NASC) and in compliance with current Good Manufacturing Practices (cGMP). Scientific evidence of efficacy should also be available in the peer-review scientific literature. Several families of supplements exist for the management of OA in companion animals. Scientific evidence is stronger for some supplements than others (65). Common veterinary supplements used for joint support and the evidence for their use is listed below.

Omega-3 Fatty Acids – Omega-3 fatty acids, also named eicosanoids, are metabolically active compounds derived from 20-carbon fatty acids, usually arachidonic acid. Lipoxygenase (LOX) and cyclooxygenase (COX) enzymes are the rate-limiting steps in the production of leukotriene B4, thromboxane (TX) A2 (TXA2), and prostaglandin E_2_ (PGE_2_). In inflammatory conditions such as OA, PGE_2_ production can be increased up to 50-fold (66). Leukotriene B4 has a potent chemotactic effect and promotes further inflammation. PGE_2_ and TXA2 promote the release of tumor necrosis factor alpha and interleukin 1β, which further promote inflammation and, in joints, stimulate the production of MMP, collagen-destroying enzymes that promotes the breakdown of articular cartilage in joints with OA. Further, PGE_2_ is a potent stimulator of pain receptors, therefore contributing to the pain resulting from OA. Eicosapentaenoic acid (EPA) can be used by COX and LOX enzymes to produce the eicosanoids PGE_3_, TXA3, and leukotriene B5, which are less active and are anti-inflammatory relative to their counterparts produced from arachidonic acid. Fish oil is a primary source of omega-3 fatty acids. The scientific evidence supporting the use of omega-3 fatty acids to alleviate the signs of OA is strong (65). In dogs with OA, a diet containing approximately 3.5% omega-3 fatty acids decreased pain and lameness, improved weight bearing, and decreased the perceived need for NSAIDs (67, 68). Approximately 480 mg/kg of fish oil (50 to 100 mg/kg of EPA) would be required as a supplement to match the amounts available in the therapeutic food discussed above (69). In a recent 16-week clinical trial in dogs with OA, fish oil supplementation (90 mg/kg EPA and 20 mg/kg DHA) added to a non-fish-based food led to improvement of indicators of pain and quality of life (70). *Perna canaliculus*, the green-lipped mussel (GLM) found in the waters around Australia and New Zealand, contains several omega-3 fatty acids: EPA, docosahexaenoic acid (DHA), and eicosatetraenoic acid (ETA). It also contains glycoproteins and glycosaminoglycans (GAG). A clinical trial in dogs with chronic OA pain reported improved mobility in dogs receiving 50 mg/kg of GLM (71).
Undenatured Type II Collagen – Undenatured Type II collagen is a form of collagen obtained through low temperature processing of chicken sternum. Several clinical studies support the safety and efficacy of undenatured Type II collagen in modulating joint discomfort resulting from OA or rheumatoid arthritis in humans. Also, several studies designed to assess the efficacy and tolerability of undenatured Type II collagen in moderating joint function and joint pain due to strenuous exercise in healthy subjects showed a positive impact. One study showed that undenatured Type II collagen decreased inflammation in healthy young dogs following strenuous exercise (72). In dogs with OA, two clinical trials investigated the effects of undenatured Type II collagen using objective measures of gait. Decreased pain was observed in these trials (73, 74). The positive impact of undenatured Type II collagen in dogs with OA appears to be dose dependent, with a stronger response noted in dogs receiving 80 mg per day than 40 mg per day (75).
Glucosamine and Chondroitin – Glucosamine and chondroitin sulphate (GCS)-based supplements were historically the most common joint supplements used in companion animals. GCS has been shown to decrease systemic inflammation in humans (76). Several systematic reviews documented their efficacy when managing knee OA (77, 78). In companion animals, GCS are safe. However, less is known about their long-term impact on cartilage health and their clinical efficacy (65, 79–81). Some studies in dogs identified benefits to receiving GCS supplements. In one clinical trial with 35 client-owned dogs, pain on palpation was lower after GCS administration (82). Other studies failed to identify clinical improvement in dogs receiving a GCS supplement (83, 84), possibly because OA has wide-ranging consequences and because GCS would not eliminate all consequences of OA. Because of their anti-inflammatory activity, GCS is likely most beneficial to puppies with early OA compared to older dogs with chronic OA.
Cannabis supplementation – Cannabis-derived extracts (phytocannabinoids) are regulated as a supplement in the United States and other jurisdictions, but they are regulated as a drug in several countries. Phytocannabinoids have emerged as a potential approach to alleviate chronic pain in humans and dogs (85). There are no known clinical studies to the efficacy and safety of the use of phytocannabinoids in growing dogs.
Fortetropin^®^ – Fortetropin is a supplement made from pasteurized, freeze dried, fertilized egg yolk. It has been shown to increase lean body mass in humans and dogs. Fortetropin has not been clinically studied for safety or efficacy in the growing dog.

In conclusion, nutraceuticals are therapeutic supplements intended to provide medical or health benefits. When managing OA, supplements can provide multiple benefits to the body through various proposed mechanisms of action. Strong scientific evidence supports the use of omega-3 fatty acids and undenatured Type II collagen.

## Pharmaceutical management of OA

The choice of medications to manage OA in dogs is based on the observed level of pain and disability. Since the perception of pain is highly individual, medical therapy should be individualized. Medical therapy is also influenced by the severity of inflammation and the chronicity of OA. Medical therapy may not fully alleviate the pain experienced by dogs with OA: pain decreases but may not be eliminated. Importantly, a treatment that may be considered a “failure” because it only partially alleviates the signs of OA, may in fact bring relief to a puppy with OA. That is why, whenever possible, the medical management of OA should include medications with scientifically proven benefits. Since a single medication often does not fully alleviate OA pain, medications with varying modes of action are routinely combined when managing OA in dogs. Medications are also combined with non-medical pain-relieving strategies such as manual therapy or electrophysical modalities (low-level heat or cold, for example) to maximize pain relief. Because the response to therapy in dogs with OA is multifaceted—affecting their limb use, mobility, willingness to exercise, playfulness, appetite, sleeping habits, and demeanor, the assessment of the response to therapy will also be multifaceted. Revaluations should include the assessment of changes in behavior and activity, observation of gait, palpation, and a client questionnaire.

Most of the research on the safety and effectiveness of medications to manage OA in puppies focused on non-steroidal anti-inflammatory drugs (NSAIDs). Consequently, NSAIDs are the most widely use pain medication in puppies with OA. In the United States, several NSAIDs have been approved for use in puppies, at ages ranging from 6 weeks to 9 months, depending on the NSAID (Table 1). In puppies whose OA pain is not fully controlled by NSAIDs, adjunctive pain medications can be considered (Table 2). However, research on medical management using medication classes other than NSAIDs is mostly absent from the peer-reviewed literature. This is in part because few clinical trials overall have included medications other than NSAIDs (79) and also because clinical trials of dogs with OA rarely focus on puppies. Practically, NSAIDs should be the first treatment choice because the available data supporting their efficacy is stronger than other pain medications. However, NSAIDs and some other drugs anecdotally used in puppies (gabapentinoids and NMDA-receptor antagonists) are primarily cleared via hepatic metabolism and immature hepatic function in puppies less than 4 to 5 months of age could result in higher serum drug concentrations, potentially leading to an increased magnitude of effects and duration of action (86). However, NSAIDs can be used down to the approved minimum age. Also, other hepatically-metabolized drugs can be administered at the low end of the dosing range, at the discretion of the prescribing veterinarian. Injectable polysulfated glycosaminoglycans have been shown to decrease inflammation and pain and to slow cartilage degeneration in puppies with hip laxity.

**Table 1:** Minimum approved age for current NSAIDs in dogs.

NSAID	Brand name, manufacturer	Minimum approved age
Carprofen	Rimadyl^®^, Zoetis	6 weeks
Firocoxib	Previcox^®^, Boehringer Ingelheim	7 weeks
Deracoxib	Deramaxx™, Elanco	4 months
Robenacoxib	Onsior™, Elanco	4 months
Meloxicam	Metacam^®^, Boehringer Ingelheim	6 months
Grapiprant	Galliprant^®^, Elanco	9 months

NSAIDs, non-steroidal anti-inflammatory drug. References: product inserts – June 2024.

**Table 2:** Medical therapy of puppies with OA.

Stage – substage	Pharmaceutical recommendation
Healthy/low risk^a^	None
Healthy/high risk^b^	Joint supplements, diet, and exercise
Already affected – mild pain	NSAIDs as needed
	Add or substitute other medications, if appropriate
Already affected – moderate pain	NSAIDs or, potentially, antiNGF mAbs
	May need to add PSGAG, amantadine, gabapentin, and other medications
Already affected – severe pain	NSAIDs or antiNGF mAbs
	Multimodal therapy with PSGAG, amantadine, gabapentin and non-medical therapy

NSAIDs, non-steroidal anti-inflammatory drugs, antiNGF mAbs, anti-nerve growth factor monoclonal antibodies, PSGAG, polysulfated glycosaminoglycans.

a Low-risk dogs are dogs with low incidence of developmental orthopedic disease, medium size, slender body conformation, and no evidence of chondrodystrophy.

b High-risk dogs are dogs with high incidence of developmental orthopedic disease, heavy conformation, large and giant size, or chondrodystrophic conformation.

While anti-nerve growth factor (anti-NGF) monoclonal antibodies are used to alleviate OA pain in adult dogs, their safety and efficacy have not been evaluated in dogs less than 12 months of age (87). The impact of anti-nerve growth factor on the developing nervous system is therefore unknown. Since the nervous system is largely mature by 6-weeks of age in puppies (88), an anti-NGF drug such as bedinvetmab could potentially be used at the veterinarian’s discretion in puppies with OA pain.

In conclusion, the perception and expression of pain is highly individual. Medical therapy should be individualized, accounting for the severity of inflammation and the chronicity of OA. NSAIDs are the main medication used to manage OA pain in puppies because of safety studies in that age group. Few clinical trials have studied the effects of medications from pharmaceutical classes other than NSAIDs and polysulfated glycosaminoglycans, but other drugs (e.g., antiNGF mAbs, gabapentinoids, and NMDA-receptor antagonists) can be used at the discretion of the veterinarian.

## Intra-articular injections in puppies

Intraarticular (IA) injections and specific surgical procedures may also be part of the management of OA. The use of IA injections is often considered when non-surgical therapy fails or in combination with some surgical procedures. These injections can be used to control symptom flares, acute episodes of exacerbation of joint pain, or to manage patients who are at increased risk of adverse event from NSAID therapy or have a history of adverse events from NSAIDs.

Intra-articular (IA) injections, also named therapeutic joint injections, have been used in humans and horses for the management of OA with acceptable safety and efficacy (89, 90). The IA products currently used for OA management in humans, horses, and dogs include regenerative medicine injections (e.g., platelet rich plasma, platelet rich factor, bone marrow aspirate concentrate, autogenous stem cells, stromal vascular fraction cells), viscosupplementation (hyaluronic acid, hydrogels), radiosynoviorthesis, and glucocorticoids (corticosteroids). IA injections have been used less commonly in dogs than in horses and human beings. Evidence of efficacy is limited to a few clinical reports and none of those focused on puppies (91). Intra-articular injections appear safe overall (92, 93). Glucocorticoids are no longer routinely used to manage OA in dogs and would not be expected to be used in puppies because they appear to accelerate cartilage breakdown in joints with OA (94).

In puppies with severe joint problems that led to OA or will lead to OA, surgical procedures should be considered before IA injections. Once a joint has been managed with surgery, or when surgery is not an option, IA therapy can be considered for its anti-inflammatory and anabolic effects on inflamed joints with early OA (Table 3). IA therapy is often considered after a surgical procedure (for example after arthroscopy) where joint disease is confirmed. For example, to manage a 6-month-old large-breed dog with elbow dysplasia, a PRP IA injection may be considered at the time of surgery and several weeks after surgery if joint effusion (a feature of inflammation) and lameness (a feature of pain) persist. Preference is given to IA injections that have potential regenerative properties (e.g., platelet rich plasma, platelet rich factor) over IA injections that have palliative properties (e.g., hydrogel, radiosynoviorthesis).

**Table 3:** Intra-articular injections in puppies with OA.

Stage/substage	Intra-articular injection products
Healthy/low risk	Not recommended
Healthy/high risk	PRP, PRP/HA combination
Already affected – mild pain	PRP, PRP/HA combination
Already affected – moderate pain	PRP, PRP/HA, PAAG
Already affected – severe pain	PRP, PRP/HA, stem cells, SVF, BMAC, PAAG

PRP, platelet-rich plasma; HA, hyaluronic acid; PAAG, polyacrylamide gel; SVF, stromal vascular fraction; BMAC, bone marrow aspirate concentrate. See Table 2 for remainder of legend.

Viscosupplementation is a type of IA injection done to increase the viscosity of synovial fluid with the intent to restore the physical properties of joint fluid. The objectives of viscosupplementation include aiding in joint lubrication, decreasing inflammation and cartilage degradation, and assisting cartilage repair (95). Viscosupplementation was initially achieved using low or high molecular weight hyaluronic acid (96). More recently, collagen elastin hydrogel microparticles (CEHM) and cross-linked polyamide hydrogels have been used (97).

Hyaluronic acid (sodium hyaluronate, hyaluronan, HA) is a fluid present in all living organisms. Intra-articular HA is a form of viscosupplementation used to manage OA in humans and horses. No study has focused on the use of HA in growing dogs with OA.

Polyacrylamide hydrogels are non-soluble, non-immunogenic, non-toxic and highly viscous 2.5% or 4% solution of cross-linked polyacrylamide in sterile water. There are no known studies in puppies, all current canine studies have included skeletally mature dogs.

## Regenerative medicine

There is increasing use of regenerative medical therapy, also named orthobiologics, in humans, horses, and dogs. Orthobiologic products include platelet rich plasma (PRP), autologous or allogenic mesenchymal stem cell (MSC) therapy, stromal vascular fraction (SVF) therapy, and bone marrow aspirate concentrate (BMAC). Safety and efficacy have been reported in humans, horses, and dogs with OA (98). Intra-articular PRP has been shown to positively impact dogs with OA (99) and promote tendon and ligament healing. Several PRP systems are commercially available and produce PRP with varying parameters (100, 101). The IA injection of PRP usually includes one to three injections weeks to months apart. To minimize the risk of negative interaction, some have recommended avoiding NSAIDs one week before to two weeks after PRP injection and avoiding cold therapy after injection. The combined use of IA PRP and HA has also been reported (102).

Stem cells are characterized by their ability to self-renew and differentiate along multiple lineage pathways, contributing to generating new tissues. Stem cells are chemotactic for progenitor cells, supply growth factors, make extracellular matrix, promote angiogenesis, are anti-apoptotic, anti-inflammatory, and anti-fibrotic (103). When used in regenerative therapy, millions to billions of cells are injected. These cells are usually autologous cells harvested using a minimally invasive procedure, differentiated along multiple cell lineage pathways in a regulated and reproducible manner, transplanted to either an autologous or allogeneic host, and manufactured in accordance with current GMP guidelines.

In dogs, autogenous sources of stem cells include bone marrow and fat. Cells are isolated, expanded, and returned to the patient. Bone marrow aspirates contain hematopoietic stem cells, which can form all types of blood cells, and MSC which can generate bone, cartilage, fat, and connective tissue. Bone marrow aspirate can be processed in-house through a series of centrifugation steps to yield BMAC (104, 105). Adipose tissue contains MSC. Adipose-derived stem cells can also be processed in-house through a series of steps involving enzymatic tissue digestion and centrifugation to obtain the SVF (106). Several clinical studies suggest that IA stem cell therapy is safe and beneficial to dogs with OA (107, 108). The combination of stem cells and PRP also has reported benefits to manage OA and for tissue healing (109–111).

The IA injection of a colloid containing a radioactive tin (tin-117 m) is commercially available to manage the signs of OA (Synovetin OA^®^). This process has not been studied in puppies, only in skeletally mature animals, where no deleterious effects to the joint were detected.

In conclusion, the goals of intra-articular therapy include improving joint lubrication, decreasing inflammation and cartilage degradation, and improving cartilage repair. IA injections can be used as an alternative to surgery in patients who are poor surgical candidates or after surgery, in patients with persistent joint effusion and pain. Studies evaluating the efficacy and long-term safety of IA in puppies are lacking.

## Surgical management of OA in puppies

Surgical procedures may be warranted to manage OA in puppies. Procedures are intended to protect joints from OA or to decrease the impact and progression of OA. In puppies, OA most often results from joint instability or joint subluxation, from the intraarticular presence of an osteochondral fragment, or from an articular fracture. Surgical procedures to manage OA in puppies aim to minimize joint instability, eliminate joint subluxation, remove osteochondral fragments, rigidly stabilize articular fractures, and potentially restore articular surfaces with autogenous or exogenous grafts (Table 4).

**Table 4:** Surgical management of OA in puppies.

Purpose of surgery	Joint	OA severity	Surgical method	Patient age
Control of laxity	Hip	Mildly affected	JPS	<20 weeks of age and DI <0.7
		Moderately affected (reduction angle ≤30^0^, subluxation angle ≤10^0^)	DPO or TPO	>6 months and < 12 months
Eliminate subluxation	Elbow		DPUO	n/a
	Stifle		Patellar luxation	Based on severity
Removal of osteochondral fragment	Elbow, shoulder stifle, tarsus		FCP, OCD, UAP	When diagnosed
Articular fracture stabilization	Humerus, femur, other			When diagnosed
	Hip		FHO, THR	Non-surgical management failure
	Shoulder		Arthrodesis	Non-surgical management failure

OA, osteoarthritis; JPS, juvenile pubic symphysiodesis; DPO, double pelvic osteotomy; TPO, triple pelvic osteotomy; DPUO, dynamic proximal ulnar ostectomy; FCP, removal of a fragmented medial coronoid process; OCD, osteochondritis dissecans; UAP, ununited anconeal process; FHO, femoral head osteotomy; THR, total hip replacement, DI, distraction index.

Canine hip dysplasia is the most common orthopedic disease in dogs. Dysplastic hip joints develop laxity after birth. Laxity is influenced by several factors, including the geometry of the hip joint, pelvic muscle mass, and the ligament of the femoral head. Subluxation results in abnormal forces being exerted at the acetabular rim and on the femoral head. The result is abnormal hip joint conformation, joint incongruity, increased hip joint laxity, and cartilage damage. During growth, pain arises from cartilage damage, subchondral bone inflammation, and joint capsule strain. In dogs with hip laxity, surgical intervention aims to increase coverage of the femoral head by acetabular rotation and lateralization to decrease femoral head subluxation.

In growing dogs between 16 and 20 weeks of age, alteration of the acetabular orientation can be achieved by limiting growth of the pubis though premature closure induced using electrocautery or mechanical crushing (112). The procedure is named juvenile pubic symphysiodesis (JPS). The JPS procedure appears to help dogs with hip laxity (113), but its outcome relative to non-surgical measures has not been evaluated in a long-term clinical trial. In dogs older than 26 weeks, alteration of the acetabular orientation can be achieved by rotating the acetabulum using a double or triple pelvic osteotomy (DPO or TPO, respectively). DPO and TPO are much more invasive than JPS and have similar clinical results. They appear to benefit dogs with hip laxity, without being curative (113). Total hip replacement (THR) is rarely done in growing dogs. A THR is recommended in dogs that fail to improve with non-surgical management and that are too severely affected to benefit from a JPS or DPO. Most of the dogs who receive a THR as puppies have luxoid hip dysplasia, a severe form of dysplasia with early dorsal luxation (114). In dogs with severe hip pain non-responsive to non-surgical management and when a THR is not considered as an option, a femoral head ostectomy (FHO) can be considered. With the FHO, the femoral head and neck are cut, and the head is removed, forming a fibrous joint. Mild pain and clinical dysfunction often persist following FHO, but dogs are often less painful once they recover from surgery.

Elbow dysplasia is an umbrella term describing several problems including radioulnar incongruity, fragmentation, or cartilage damage to the medial coronoid process. Elbow dysplasia is most common in large-breed dogs. It starts during growth and is commonly diagnosed between 6 and 10 months of age. The medial aspect of the elbow joint is more vulnerable to OA secondary to elbow dysplasia than the lateral aspect of the joint. Some dogs have osteochondritis dissecans (OCD) of the medial aspect of the humeral condyle. Elbow dysplasia is managed conservatively or with surgery. Surgical procedures include the removal of osteochondral fragments form the medial coronoid process or humeral condyle, potentially combined with an ulnar osteotomy (bi-oblique ulnar osteotomy) to improve joint congruity.

Corrective osteotomies that alter the direction of forces traveling across the elbow joint and increase loads resisted by central and lateral aspects of the joint have been reported, including the sliding humeral osteotomy and the proximal abducting ulnar osteotomy. The long-term benefits of these surgical procedures are unclear. Most dogs with elbow dysplasia develop OA (115), which progresses over time. Focal joint resurfacing and partial or total elbow replacement may be considered in dogs with severe chronic lameness non-responsive to conservative management or other surgical procedures. In some dogs, the anconeal process, which develops from a secondary center of ossification that normally ossifies with the ulna at approximately 6 months of age, does not fuse with the ulna, leading to an ununited anconeal process. Surgery is recommended for management. Non-displaced processes can be managed with a bi-oblique ulnar osteotomy. Displaced processes can be reduced and secured to the ulna with a bone screw and protected with a bi-oblique ulnar osteotomy. Deformed anconeal processes are excised.

Perthes disease is a developmental problem affecting the femoral head and hip joint. With Perthes disease, growing small-breed dogs experience a collapse of their femoral head presumably resulting from damage to the blood supply to the proximal femoral epiphysis. The collapsed femoral head, visible on a ventrodorsal radiograph, is a source of joint pain and OA. Diagnosis may be delayed by weeks to months, possibly because owners and veterinarians may not anticipate the presence of hip joint problems in small breed dogs. The non-surgical management of OA secondary to Perthes disease is implemented and surgery (THR or FHO) is recommended for patients that do not respond to conservative management.

Patellar luxation is a developmental problem secondary to skeletal abnormalities to the femur and tibia that alter the direction of forces exerted by the quadriceps femoris muscle on the patella. Early luxation of the patella may further alter the growth of the distal portion of the femur and proximal portion of the tibia, and, in severe cases, can lead to a loss of stifle joint extension. Luxations are often medial but can be lateral. The asymmetric forces generated by the luxated patella on the physes, particularly on the distal femoral physis, may lead to increased growth in the side under tension and decreased growth on the side under compression (Hueter-Volkmann law) (116). Luxations that occur earlier in life likely result in more severe displacement, growth abnormalities, and tissue changes than luxation that occur near adulthood. Patella luxation has been classified as grade 1 to 4. With grade 1, the patella can be luxated with digital pressure and returns when released. With grade 2, the patella can be luxated and does not return immediately when released. With grade 3, the patella is found luxated but can be reduced to its normal position. With grade 4, the patella is luxated and cannot be reduced.

Mild forms of patellar luxation may be manageable without surgery. Severe forms of patellar luxation require surgery. Most small-breed dogs with patellar luxation have OA at the time of presentation for surgery, and the severity of OA is greater when the dogs are older (27), suggesting that OA progresses even when patellar luxation is managed conservatively.

The objectives of surgery most often include deepening the trochlear groove and aligning the quadriceps femoris with the trochlea. This can be achieved with a range of methods whose complexity are proportional to the severity of the problem. Dogs that develop grade 4 patellar luxation early in growth may lose stifle joint extension, because the quadriceps no longer acts as an extensor of the stifle. These dogs need surgery as early as possible to prevent further loss of extension and to restore limb use.

Cranial cruciate ligament injury is common in dogs and sometimes occurs in puppies. Subjectively, the problem is most prevalent in working and sporting dogs that begin training at 5 or 6 months of age, before skeletal maturity (117). The ligament can tear or can avulse at its femoral origin or tibial insertion. The diagnosis of a partial or complete cranial cruciate ligament rupture may be achieved using arthroscopy. Small diameter scopes (< 2 mm) are increasingly used to diagnose these injuries. Dogs with (minor) partial ligament tears may be managed with an intraarticular injection of PRP, combined with rest. Dogs with cranial cruciate ligament injury are often managed with surgery and most of them have OA at the time of presentation (27, 118). Osteoarthritis in dogs with cranial cruciate ligament injury is more severe than in dogs with patellar luxation, possibly because cranial cruciate ligament injury is more inflammatory than patellar luxation. Several surgical procedures are used to manage cranial cruciate ligament injury, particularly tibial plateau leveling osteotomies. It may be possible to repair some cranial cruciate ligament avulsions. Some surgical procedures interfere with the proximal tibial growth plate and should probably be avoided in puppies.

Osteochondral fragments occur in joints because of osteochondritis dissecans (OCD). These fragments are found on the axial aspect of the lateral femoral condyle in the stifle joint and in the shoulder, elbow, and tarsus. The condition is identified most often in male, large, or giant dog breeds between 6 and 10 months of age. Osteochondral fragments lead to pain, lameness, and OA. Dogs that do not respond to conservative management undergo surgery. Historically, the bone fragment was removed from the joint. This appears to work well in the shoulder joint. Alternative procedures such as osteochondral autograft or synthetic (metal-backed polyurethane implants) have emerged. In the tarsus, OCD fragments often involve the proximal-central zone of the medial ridge of the talus. Most joints with OCD develop OA. Some dogs, generally small-breed dogs, have glenoid dysplasia, an abnormal development of the glenoid articular surface, possibly secondary to laxity or luxation early in growth. Conservative management may not help dogs with joint luxation. An arthrodesis of the shoulder joint may be recommended.

Conformational abnormalities are common in dogs, particularly deformities of the antebrachium. Most conformational abnormalities result from genetic developmental disease such as chondrodystrophy or chondrodysplasia (52). Abnormal growth of the distal ulnar physis leads to a curvature of the radius and elbow incongruity. The problem affects both forelimbs. In contrast, unilateral deformities generally result from an injury to a growth plate of the ulna or radius. Radial head subluxation or luxation can occur. Mild cases are monitored and managed conservatively. Surgery may be recommended to manage severe problems. Corrective osteotomies can decrease angulation and torsion, restore limb length, and improve joint subluxation. In one report, half of the dogs with antebrachial deformities had OA at the time of presentation (119). In that report, the presence of OA and the severity of lameness at the time of presentation were factors that negatively impacted the outcome of surgery, suggesting that antebrachial deformities should be managed without delay in puppies, before the onset of OA.

In conclusion, OA most often results from joint instability or joint subluxation, from the intraarticular presence of an osteochondral fragment, or from an articular fracture. The purpose of surgical procedures is to protect joints from OA or to decrease the impact and progression of OA.

## Case examples

### 6-month-old male Labrador retriever with hip dysplasia

A 6-month-old male Labrador retriever presents with hip dysplasia diagnosed based on a ventrodorsal radiograph that shows bilateral hip subluxation and mild osteophytosis. Upon observation, the dog has swaying gait in pelvis limbs. On palpation, a moderate pain response to extension of both hip joints is observed. The owner considers surgical options. The dog is too old for a JPS and not sufficiently painful or debilitated for a DPO, THR, or FHO. The initial management of OA is conservative. The owner fills out a baseline CBPI questionnaire, which shows minimal pain severity and pain interference. An NSAID is selected as baseline pain medication and prescribed as needed (PRN). See Table 1 for minimal age for various NSAIDs. Growth and weight optimization are discussed. Nutritional advice includes continuing with large breed puppy food and maintaining an optimal body condition of 5/9. A decision to postpone castration until a year of age is made to protect the dog against weight gain after castration. Daily walks are recommended and an exercise program of 30-min leash walks four times a week is started. Nutritional supplementation with omega-3 fatty acids and Undenatured Type II collagen is implemented. The anticipated signs suggestive of symptom flares and OA progression are discussed with the owner. The owner is tasked to keep a log of OA signs, including the activities preceding any increase in clinical signs. A reevaluation after 3 months is scheduled, with the understanding that the dog will be reevaluated sooner if clinical signs increase. Strengthening exercises are discussed, including weight-shifting, sit-to-stand, and backward walking exercises.

### 6-month-old female French Bulldog with elbow subluxation and OA

A 6-month-old female French Bulldog presents with a forelimb lameness and OA of both elbow joints, diagnosed on mediolateral radiographs of the elbow joint that show humero-ulnar subluxation and mild osteophytosis. Upon observation, the dog is hesitant to walk down steps. When resting, the dog flexes his elbow and wrist joints, possibly to relieve pain. On palpation, a mild pain response to full flexion of the elbow joint is present. A computed tomography scan is discussed to rule out the presence of coronoid process fragmentation, delayed ossification of the humeral condyle, and other abnormalities potentially invisible on radiographs. The owner fills a baseline CBPI questionnaire. Ovariohysterectomy is discussed and scheduled. The importance of maintaining a lean body condition is discussed. Surgical options, including arthroscopy and DPUO, are discussed. The owners select conservative management. An NSAID is prescribed after baseline bloodwork. An exercise program is initiated with 10- to 15-min-long leash walks done twice daily, whenever possible based on weather and owner availability. A reevaluation is planned after 30 days, unless clinical signs warrant an earlier evaluation.

### 9-month-old male Bernadoodle with elbow dysplasia

A 9-month-old castrated male Bernadoodle presents with elbow dysplasia diagnosed based on mediolateral radiographs. A baseline CBPI is filled by the owners, indicating mild severity and minimal interference. The dog’s body condition is rated as slightly overweight (6/9). The dog has a mild lameness of his forelimbs resembling a slightly hypermetric gait, suggesting elbow pain. On palpation, a mild pain response to flexion of the elbow joints is observed. A weight loss program to reach optimal body condition (5/9) is initiated. Nutritional supplements (omega-3 fatty acids from fish oil and Undenatured Type II collagen) are recommended. An exercise program based on leash walks and gentle play is initiated. Exercises are initiated, including down-to-up, waving, high-five, and weight-shifting, with a target of 20 min every other day at the beginning to be increased to 30 min per day after 1 month. Several sessions of weekly hydrotherapy, with underwater treadmill walk and swim, are prescribed.

## Integrating all approaches to manage OA in puppies

Puppies commonly have OA. The most common causes of OA that develop during the first year of life appears to be hip dysplasia (120), elbow dysplasia, and patellar luxation. Injuries to the cranial cruciate ligament are relatively uncommon in puppies. Few surgical procedures have been shown to positively impact the course of OA over a lifetime and, to be maximally protective, these procedures should ideally be done before the development of OA. Since the development of OA is rapid and often insidious, protective surgical procedures are rarely done and most growing dogs with OA are managed conservatively.

Despite how common OA is in puppies, the management of OA in growing dogs has not received much coverage in the scientific literature. That dearth of literature results from the fact that the impact of OA in growing dogs is less severe than in older dogs. In growing dogs, OA mostly leads to acute pain and in adult dogs OA leads to chronic pain combined with loss of muscle mass, joint motion, and fitness and potentially leads to behavioral changes. Subjectively, puppies with OA may also show mild behavioral changes, they may be more introverted and less playful than puppies without OA. This suggests that OA could negative impact socialization and training. Further research is warranted on that issue.

Because OA management will be required over the dog’s lifetime, it is often daunting to owners and clinicians to work-up, discuss, and manage OA when it is diagnosed in a puppy. It is important to prioritize management options that does not overburden or overwhelm owners (financially or logistically) in the short term and are sustainable in the long term (Table 5). Safe and effective management that are affordable and convenient are prioritized. Beyond the initial management plan, a management plan for symptom flares should also be developed early so that owners are prepared to deal with periodic recurrences of clinical signs. Symptom flare plans generally include the identification of flare triggers, rest, and anti-inflammatory and pain-relieving steps. To ensure the short-term and long-term success of OA management, communication between owner and the clinical team should be simple and sustained (53). Therapy should be individualized to the needs of the patient and the preferences of the owner (53). The efficacy of OA management steps is particularly important when treating early OA in puppies because management steps with minimal efficacy are unlikely to be sustained over the long term by owners. Also, ineffective therapy may compromise owner trust and could endanger the long-term relationship that is required to manage OA over a lifetime.

**Table 5:** Prioritizing OA therapeutic options in puppies.

Whole-body wellness	Physical rehabilitation	Supplements	Medications	IA injection	Surgery
Healthy/low risk					
Weight^a^ management	Controlled exercise^b^	Omega-3 fatty acids^b^	NSAIDs^b^ (PRN)	None	None
Nutrition^a^		Undenatured type II collagen^b^			
Healthy/high risk					
Weight^a^ management	Controlled exercise^b^	Omega-3 fatty acids^a^	NSAIDs^b^ (PRN)	PRP/HA^b^ (combined)	Surgical correction^a^
Nutrition^a^		Undenatured type II collagen^a^	Amantadine, gabapentin^b^	PAAG	None
Already affected					
Weight^a^ management	Controlled exercise^b^	Omega 3^a^ fatty acids	NSAIDs^b^ (PRN)	PRP/HA^b^ (combined)	Salvage procedure
Nutrition^a^		Undenatured type II collagen^a^	PSGAG, amantadine, gabapentin^b^	PAAG^b^	

a Primary therapeutic focus.

b Secondary therapeutic focus.

See Table 3 for remainder of legend.

While OA is described as a progressive disease, few studies have evaluated the progression of OA over time (53). There is a lack of knowledge about how rapidly OA might transition in dogs from a physiologic problem to a physical problem, and to a functional disability. The speed of these transitions appears to vary based on the cause of OA, its initial severity, the number of joints and limbs involved, the dog size, demeanor, body condition, and living conditions (53, 121). Based on changes in osteophyte size, the progression rate of radiographic OA is slow (53). In one study, progression of radiographic OA was more rapid in puppies than adults and in spayed females (121). However, the correlation between the size of osteophytes and joint inflammation in dogs with OA appears to be poor (122). In other words, some dogs have mild radiographic signs of OA but have severe clinical signs and other dogs have severe radiographic. OA but show few clinical signs and have no functional impairment. The progression of pain is most relevant for dogs with OA. The pain resulting from OA also varies widely among dogs based on the cause of OA, its initial severity, the number of joints and limbs involved, the dog size, demeanor, body condition, and living conditions (51). While puppies are vulnerable to trauma, counterintuitively in one studies of large-breed puppies, dogs with hip dysplasia showed fewer clinical signs when they engaged in off leash activity early in life than when they did not have off leash activity (123).

The primary management goals of OA in puppies are to alleviate pain and decrease joint inflammation. Little is known about the inflammatory burden of various forms of OA in dogs. For dogs with chronic hip dysplasia, inflammation is present in the joint capsule, joint fluid, and subchondral bone (124). The fact that OA has an impact that varies widely among dogs suggests differences in inflammation. For example, among dogs presenting for the surgical management of patellar luxation and cranial cruciate ligament injury, OA from cruciate ligament rupture is more severe than OA from patellar luxation, suggesting that OA from cruciate ligament rupture carries a heavier inflammatory burden (27). Pain relief in puppies is often achieved using NSAIDs, rest, and low-intensity exercise. Other therapeutic goals include being able to achieve proper socialization and training and maintaining limb strength. Optimizing growth, avoiding excess body weight, and cautiously timing neutering are also priorities. The extent and complexity of OA management in puppies should be based on severity of the problem and initial response to therapy. Therapy is often introduced in phases with an initial focus on nutrition, nutritional supplementation, and activity oversight, followed by an NSAID and other medications, followed by joint injections, followed by a surgical procedure. Salvage surgical procedures can have a transformative impact on painful joints with OA in puppies, but they are used as a last resort. Osteoarthritis management programs are individualized. They incorporate owners’ therapeutic philosophy and goals. They take into account the dog’s demeanor and limitations (53). Because individual results vary with all types of therapy, objective reevaluations are critically important. Clinicians should be mentally prepared to change therapeutic course based on patient response to therapy and disease progression. The detection and management of symptom flares is also important. The source of flares should be identified whenever possible. In puppies with OA, good days should vastly outnumber bad days.

In conclusion, OA often affects growing dogs. Its clinical signs often result from joint inflammation and acute pain. The early diagnosis of OA in growing dogs can be challenging because changes are less severe than in older dogs. The management of OA in growing dogs relies on whole body wellness, nutritional oversight and supplementation, medications, electrophysical physical modalities, and exercise. Intra-articular injections and regenerative medicine are used less often in growing dogs than in adult dogs in part because simpler measures are often sufficient to control clinical signs and less is known about their impact in growing dogs than in adult dogs. Surgery is used in growing dogs to improve the long-term joint health or manage pain that cannot be controlled with non-surgical steps. To be sustainable in the long term, the early management of OA in growing dogs should carry a low practical and financial burden for owners, in addition to being safe and effective.
