# Correction: A proposed framework for practical multimodal management of osteoarthritis in growing dogs

**DOI:** 10.3389/fvets.2026.1801648

**Published:** 2026-02-26

**Authors:** Denis J. Marcellin-Little, Donald A. Hulse, Janice L. Huntingford, Tamara Grubb, Matthew W. Brunke, Arielle Pechette Markley, Bethany Frank

**Affiliations:** 1 Department of Surgical and Radiological Sciences, School of Veterinary Medicine, University of California, Davis, Davis, CA, United States; 2 College of Veterinary Medicine, Texas A&M University, College Station, TX, United States; 3 Essex Animal Hospital, Essex, ON, Canada; 4 Department of Veterinary Clinical Sciences, Washington State University, Pullman, WA, United States; 5 Veterinary Referral Associates, Gaithersburg, MD, United States; 6 Red Sage Integrative Veterinary Partners, Fort Collins, CO, United States; 7 Vetoquinol United States, Fort Worth, TX, United States

**Keywords:** osteoarthritis, dog, physical rehabilitation, weight management, non-steroidal anti-inflammatory drugs, nutraceuticals, nutritional supplements, medical management guidelines

In the published article, there was a mistake in Table 1 as published.

**Table 1:** Minimum approved age for current NSAIDs in dogs.

**NSAID**	**Brand name, manufacturer**	**Minimum approved age**
Carprofen	Rimadyl^®^, Zoetis	6 weeks
Firocoxib	Previcox^®^, Boehringer Ingelheim	7 weeks
Deracoxib	Deramaxx™, Elanco	4 months
Robenacoxib	Onsior™, Elanco	4 months
Meloxicam	Metacam^®^, Boehringer Ingelheim	6 months
Grapiprant	Galliprant^®^, Elanco	9 months

NSAIDs, non-steroidal anti-inflammatory drug. References: product inserts—June 2024.

The manufacturer of Onsior™ was erroneously listed as Boehringer Ingelheim. The correct manufacturer of Onsior™ is Elanco.

The corrected Table 1 appears below.

The original version of this article has been updated.

